# A Randomised Controlled Trial to Delay or Prevent Type 2 Diabetes after Gestational Diabetes: Walking for Exercise and Nutrition to Prevent Diabetes for You

**DOI:** 10.1155/2015/423717

**Published:** 2015-05-18

**Authors:** A. S. Peacock, F. E. Bogossian, S. A. Wilkinson, K. S. Gibbons, C. Kim, H. D. McIntyre

**Affiliations:** ^1^School of Nursing and Midwifery, The University of Queensland, Brisbane, QLD 4067, Australia; ^2^Mater Research Institute, The University of Queensland, Brisbane, QLD 4101, Australia; ^3^Mater Health Services, Brisbane, QLD 4101, Australia; ^4^University of Michigan, Ann Arbor, MI 48109, USA; ^5^School of Medicine, The University of Queensland, Brisbane, QLD 4067, Australia

## Abstract

*Aims*. To develop a program to support behaviour changes for women with a history of Gestational Diabetes Mellitus (GDM) and a Body Mass Index (BMI) > 25 kg/m^2^ to delay or prevent Type 2 Diabetes Mellitus. *Methods*. Women diagnosed with GDM in the previous 6 to 24 months and BMI > 25 kg/m^2^ were randomized to an intervention (I) (*n* = 16) or a control (C) (*n* = 15) group. The intervention was a pedometer program combined with nutrition coaching, with the primary outcome increased weight loss in the intervention group. Secondary outcomes included decreased waist and hip measurements, improved insulin sensitivity and body composition, increased physical activity, and improved self-efficacy in eating behaviours. *Results*. Median (IQR) results were as follows: weight: I −2.5 (2.3) kg versus C +0.2 (1.6) kg (*P* = 0.009), waist: I −3.6 (4.5) cm versus C −0.1 (3.6) cm (*P* = 0.07), and hip: I −5.0 (3.3) cm versus C −0.2 (2.6) cm (*P* = 0.002). There was clinical improvement in physical activity and eating behaviours and no significant changes in glucose metabolism or body composition. *Conclusion*. A pedometer program and nutrition coaching proved effective in supporting weight loss, waist circumference, physical activity, and eating behaviours in women with previous GDM.

## 1. Introduction

Gestational diabetes mellitus (GDM) is a well-established predictor for the development of type 2 diabetes (T2DM) [1]. The incidence of GDM has been increasing over the last fifteen years [2], and, with the introduction of updated clinical guidelines for the diagnosis and management of GDM, the prevalence in Australia could be as high as 13% [3]. Worldwide, the prevalence of T2DM following GDM may be as high as 70% [4–9].

In 2007, the economic burden of T2DM was estimated at approximately $US218 billion [10]. The global burden of T2DM is immense [11] with one potential solution being a targeted delay or prevention of progression to T2DM in high risk populations [12–15]. However, programs designed to target women following GDM have met with varied levels of success [16]. Lifestyle intervention trials incorporating dietary modification and promoting increased physical activity to support weight loss have been successful in preventing T2DM [16–19], demonstrating a reduced risk of progression to T2DM in high risk groups by up to 58% [20, 21], with a continuing influence up to eight years after the intervention [22].

In a secondary analysis of the US Diabetes Prevention Program study, women with documented prior GDM had a 71% greater chance of progressing to T2DM three years later, a risk which was reduced by 50% through lifestyle intervention [18]. However, women were over a decade from their delivery, and it was not known whether this last delivery was in fact their GDM delivery. Therefore, although interventions successfully reduced the incidence of diabetes, the onset of diabetes likely occurred after subsequent pregnancies. Surveys of women with GDM suggest that six months–two years is an optimal time to offer a lifestyle modification intervention as women felt they would be more able to include changes in their life after the birth of their baby [23], and earlier intervention would also offer the chance to reduce the risk of glucose intolerance during subsequent pregnancies. Targeting these reproductive-aged women with recognised risk factors with programs that both engage and provide education for long-term healthy behaviour may provide the optimal prevention strategy for both maternal and fetal outcomes.

A recent systematic review examined types of physical activity and found the most successful exercise programs in postpartum women were those with objectively set goals usually incorporating devices such as pedometers [24]. Previous studies that specifically used pedometers in the postpartum population report an increase in physical activity [25, 26]. Both studies relied on self-reporting of step counts from the pedometer, with no indication as to whether the women would have preferred web-based storage of the step data. Kim et al. suggested the combination of internet based support with a more traditional approach may be more successful than the internet support alone [27].

## 2. Objectives

This study aimed to develop, implement, and evaluate a low intensity exercise and diet program for women who were diagnosed with GDM during a prior pregnancy and had a body mass index (BMI) > 25 kg/m^2^ in the postpartum period. Our primary hypothesis was that the women in the intervention group would achieve significantly more weight loss than the control group. Our secondary hypotheses were that, compared with women in the control group, women in the intervention group would have significantly (1) better diet quality and self-efficacy, (2) more minutes of physical activity/week, (3) lower fasting glucose and insulin levels, and (4) lower body fat mass (FM) and significantly higher fat free mass (FFM). The trial was named “walking for exercise and nutrition to prevent diabetes for you” (WENDY).

## 3. Method

The intervention took place at a tertiary maternity hospital in Brisbane, Australia, from June 2011 to December 2012. The study was approved by Mater Health Services Human Research Ethics Committee and The University of Queensland Medical Research Ethics Committee.

We evaluated the intervention using a randomised controlled trial. Women were eligible if they were 18 years of age or over and had been diagnosed and treated for GDM, six months to two years postpartum, had a self-reported BMI > 25 kg/m^2^, had routine access to a computer, computer skills to navigate websites, and e-mail, and understood that the primary physical activity would be walking. Women were ineligible if they were currently pregnant, had T2DM, were not fluent in English, used hypoglycaemic medications, or had any mental or physical disabilities which would have hindered participation in study activities. Randomisation was stratified according to BMI (25–30 kg/m^2^; >30 kg/m^2^).

Women were recruited through several venues, including telephone contact obtained from the hospital database of women with GDM diagnoses, hospital-based electronic resources, advertisements placed through the Australian National Diabetes Services Scheme (NDSS) [28] dedicated website to GDM (You2), and television advertisements.

Participants were contacted by the research team, with three attempts at contact (fixed and mobile phones). Women not contactable after three attempts were classified as “unable to contact.” Those who were contacted and refused had their reasons for refusal noted. For those who agreed to participate, an e-mail address and basic data such as height and weight to allow calculation of current BMI and updated contact details were collected, and an oral glucose tolerance test (OGTT) was performed to exclude T2DM.

## 4. Randomisation

An independent service generated a stratified, variable block, computer generated randomisation schedule and sealed the individual allocations in opaque envelopes. The envelopes were stored in a locked, secured container until eligibility was established. Once eligibility was established through baseline measurements (BMI, no T2DM on OGTT), the next envelope for the appropriate stratum was opened.

Women allocated to the intervention group received a pedometer linked to a tailored web-based program “step up to health” and a four-week nutrition coaching workshop. The women in the control group formed a wait-list group and were offered the nutrition workshop following the three-month assessment.

The pedometer had an opaque sticker that covered the digital display and was worn continuously for the first week, without providing feedback to record baseline steps. Once the baseline steps were uploaded via USB, the sticker was removed and the step count was visible. The web-based program generated weekly goals based on the previous weeks steps. As the steps were uploaded each week, the goals were gradually increased, until the maximum of 10,000 steps/day was reached [27]. The user was encouraged to log on weekly to receive updated weekly goals, feedback on their walking progress, messages, and “tips” regarding diet and exercise targeted at diabetes prevention.

The nutrition coaching workshop was delivered by accredited practising dietitians. The workshop consisted of four one-hour group sessions incorporating evidence-based strategies to facilitate behaviour change aimed at healthy sustainable weight loss [29] and to build self-efficacy such as goal setting and self-monitoring and use of group activities to model recommended behaviour and engender peer support. Resources provided to all women included tools designed to encourage portion control [30, 31].

## 5. Data Collection and Outcome Measures

Data were collected at baseline and three months. Baseline observations included survey-based assessments of dietary and physical activity, mental health assessments, assessments of anthropometrics, body composition, serum insulin, and OGTT performance. Weight was measured to the nearest 0.1 kg using a spring balance scale, and height was measured with a wall mounted stadiometer to the nearest 0.5 cm. Hip and waist measurements were taken with a standard tape measure, and estimation of body composition (fat mass and lean body mass) was assessed using using a multifrequency bioelectrical impedance analyser (BodyStat 1500MDD, Bodystat, United Kingdom), with a measured resistance at a fixed frequency of 50 Hz.

Dietary quality was assessed using the Fat, Fibre Index [32], eating behaviour self-efficacy was assessed using The Health and Wellbeing Self Efficacy Survey (WEL) [33], physical activity was assessed using Australian Women's Activity Survey (AWAS) [34], and mental health was assessed using the Kessler Psychological Distress scale (K10) [35]. Any results indicative of anxiety or depression were discussed with the participant and referred to relevant health care providers if necessary [36]. The homeostasis model assessment of insulin resistance (HOMA-IR), a widely used estimate of insulin resistance in the fasting state, was calculated as fasting plasma insulin (FPI)-[mU/L] × fasting plasma glucose (FPG) [mmol/L]/22.5 [37].

## 6. Outcome Measures

The primary outcome was weight loss from baseline to three months, reported as absolute weight loss for each participant.

Secondary outcomes were change in measurements from baseline to three months for (1)  hip and waist measurements, (2) diet quality measured by a self-reported survey, (3) WEL overall and domain scores, (4) minutes of physical activity/week (as health enhancing physical activity, HEPA), (5) glucose and HOMA-IR, and (6) body FM and FFM.

## 7. Statistical Methods

Analysis was by intention-to-treat with all analyses comparing the control and intervention groups. Analysis was undertaken with blinding to study assignment.

Data were checked for normality of the distributions of continuous variables. Normally distributed variables underwent parametric analyses; continuous non-normally distributed data were analyzed using nonparametric methods and categorical data were analyzed using chi-squared or Fisher's exact test. Analysis of the primary outcome used independent samples *t*-test, examining percentage of weight loss between the control and intervention groups. Analyses were performed in SPSS version 15 [38]. Results are reported as mean (standard deviation [SD]) or median (interquartile range [IQR]).

## 8. Results

Demographic and anthropometric characteristics of the study participants were similar in each group (Table 1). There were more multigravidas in the intervention group, and an equal proportion of women had public and private health insurance. Ethnicity was predominately Caucasian women, with three women of Asian descent. The majority of women had required insulin therapy (control *n* = 10 [67%], intervention *n* = 9 [56%]) to control their glucose levels during pregnancy, followed by diet (control *n* = 4 [27%], intervention *n* = 4 [25%]) and then metformin (control *n* = 1 [7%], intervention *n* = 3 [19%]).

**Figure 1:**
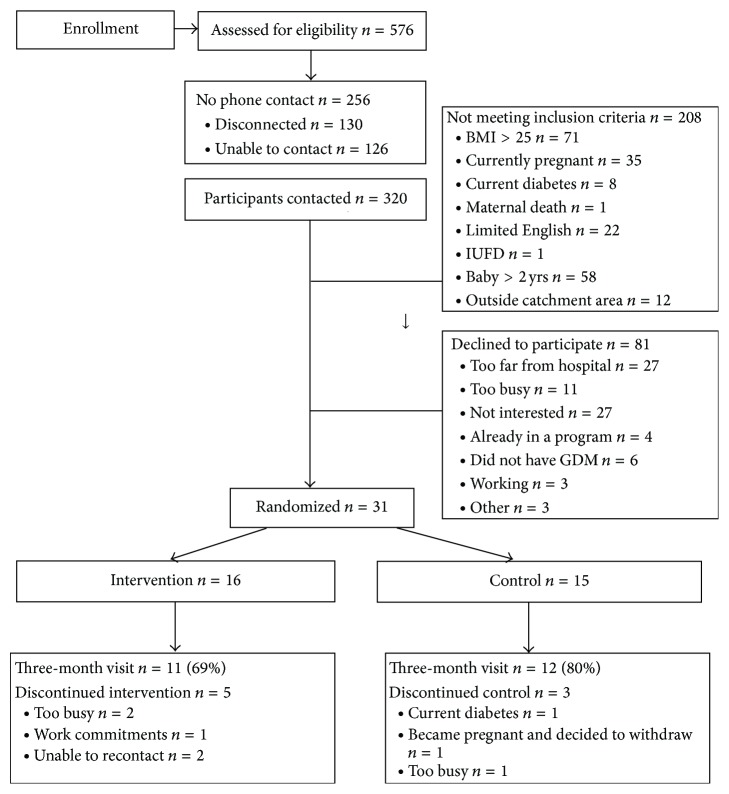
Consort diagram of the study.

We attempted to contact two hundred and forty-six women (Figure 1). Thirty-one women were randomised, with twenty-three women completing the three-month primary outcome measurements.

Five participants in the intervention group discontinued over the course of the 3-month period for differing reasons (Figure 1). One control participant (who was randomised in error prior to OGTT results) was diagnosed as T2DM following baseline OGTT and two other participants withdrew for unspecified reasons. Eleven participants in the intervention group (69%) and 12 participants in the control group (80%) completed both baseline and three-month assessments.

Weight loss was greater in the intervention group, with a median loss of 2.5 kg (1.4) compared with a static weight in the control group (*P* = 0.002), leading to a reduction in BMI of 0.9 kg/m^2^ (IQR 0.7) (*P* = 0.002) in the intervention group (Table 2).

## 9. Secondary Outcomes

Changes in hip circumference were also significant with a median loss of 3 cm (5.0) in the intervention group compared with 0 cm (4.8) (*P* = 0.006). Intervention group waist circumference decreased by a median of 3 cm (4.0) compared with 0.5 cm (4.8) (*P* = 0.037).

There was a slight decrease in body fat and increase in lean body mass in the intervention group, but this was not statistically significant. Fasting glucose taken at both data collection points showed a small difference between the two groups that had borderline statistical significance (*P* = 0.052); however, there was no change in HOMA-IR.

The intervention group increased their daily activity by one hundred and thirty-five minutes/week at the three-month time point compared to the control group, although this difference was not statistically significant. The WEL results showed participants in the intervention group at three months feeling empowered when presented with opportunity for poor food choices (*P* = 0.036). Despite being not statistically significant, trends towards improvements in the domains of negative emotions, social pressure, physical discomfort, and positive activities were noted, all related to the participants' feelings regarding food and food choices (Table 2). There were no differences in factors related to depression or mood changes between groups.

## 10. Website “Stepping up to Health”

All women randomised to the intervention group accessed the website during the three-month intervention. The mean number of participant pedometer uploads was 90 (SD 31). The mean recorded steps/day were 5,916 (SD 2,878, range 5–16,645) in the three-month period (Table 3).

## 11. Discussion

Although women with GDM are at increased risk for diabetes and a significant proportion will develop T2DM within the decade after their GDM delivery, interventions successfully targeting women during this time are few. In this study, we demonstrate that a simple, brief intervention consisting of only 4 sessions of counseling and a web-based activity component could successfully reduce weight, increase physical activity, and improve constructs associated with improved lifestyle behaviours. Such a program has the potential to be delivered in multiple care settings for limited cost. However, our study also demonstrates the challenges of engaging women with young children in an intervention aimed at changing lifestyle behaviours, as willingness to participate in the relatively “simple” intervention was low.

Obesity is a primary risk factor for the development of T2DM [1]. At least two systematic reviews [39, 40] have suggested that a combination of diet and exercise rather than diet alone may be more efficacious for postpartum weight loss [24, 39, 40]. A previous report using only the web-based pedometer component targeting physical activity did not demonstrate significant weight loss [27], suggesting that both diet and exercise components are necessary, even though we did not note significant changes in dietary quality. Of note, the pattern of clinically significant changes in physical activity with smaller, nonsignificant diet quality changes was also observed in recent dietary and physical intervention underpinned by similar behaviour-change strategies for high BMI women in the postpartum period [41]. The results for the secondary outcomes in our study such as the trend of increased incidental activity and improved self-efficacy in food behaviour in the intervention group may be a collateral effect of goal setting behaviour. The value of increased physical activity in all domains is an important factor in overall lifestyle change.

Our study also suggests that an in-person counseling component may be more effective for behaviour change in this specific at-risk group of women than the web-based program alone. The mean attendance in the four counseling sessions was three (range 0–4 sessions) (Table 3) with the majority of participants attending all four group sessions. These results suggest that the primary impact of the intervention was mediated through the in-person counseling session. Other interventions targeting obesity and risk reduction of T2DM have noted that behaviour may be successfully modified by counseling sessions only [17], but participant populations in those studies were older and had different motivators and enablers of behaviour change.

Recruitment of participants in the early postpartum phase has been proven to be difficult. Although we demonstrated promising weight and behaviour changes amongst participants, it is also notable that the participation was low and needed extensive advertising and outreach to obtain the small numbers enrolled in this study. Common themes encountered by other intervention studies in this population such as lack of time, no childcare, and difficulties “fitting the changes” into the family were also a factor in this study and affected all stages of the project from recruitment of possible participants and attrition during the trial to poor followup attendance [27, 42, 43]. While the intervention was designed to reduce barriers to behaviour change, this experience suggests that additional motivators will need to be explored in order to successfully change behaviour in this group of young mothers.

The strength of our study lies in the physical and lifestyle changes achieved in the intervention group of our sample. The feedback from the participants on the combination of the pedometer and website was positive and the delivery and content of the nutrition workshop were well received. The ability to provide the intervention in a central location was also a strength as most women found the hospital a familiar environment.

There were limitations in this project. Despite our efforts to recruit a larger number of participants, actual recruitment was low; therefore, the statistical power to detect significant differences between intervention and control arms was limited. Moreover, the women in this study were predominantly Caucasian and in their mid-thirties, and thus our results may not apply to women of other age or racial/ethnic groups. Younger women may have lower perceptions of risk and less motivation to alter behaviour [44, 45], and women of other races/ethnicities may have different perceptions and understanding of lifestyle changes required to decrease their risk of developing T2DM [46].

## 12. Conclusion

Despite encountering similar barriers to recruitment and retention of participants as in other intervention trials, results from this study demonstrate that the combination of a web-based pedometer intervention with nutrition coaching underpinned by behaviour change theory based on long-term behaviour change can lead to overall weight loss and increased physical activity (known risk factors for the development of T2DM) over a three-month period. The availability of a program that combines these features in a suitably delivered format to engage women previously diagnosed with GDM in a larger scale trial may delay or prevent T2DM in this high risk group.

This trial is registered with Australia and New Zealand Clinical Trials registry ACTRN12611000075987.

## Figures and Tables

**Table 1:** Demographic characteristics of women in intervention and control groups.

Characteristic	Group	Total *N* = 31
Age at OGTT^*^ (years)		36.0 (4.5) Range 28–44

Ethnicity	Caucasian	28 (90%)
	Other	3 (10%)

Health insurance status	Public (NHS)	17 (55%)
	Privately funded	14 (45%)

Gravidity	1	6 (19%)
	2+	25 (81%)

Parity	0	6 (19%)
	1+	25 (81%)

Diabetic control with GDM	Insulin	19 (61%)
	Metformin	4 (13%)
	Diet	8 (26%)

Weight^*^ (kg)		85.7 (17.5)

BMI^∧^ (kg/m^2^)		30.3 (8.2)

Waist^*^ (cm)		100.7 (11.8)

Hip^*^ (cm)		116.6 (14.1)

Body fat %^*^		37.4 (7.1)

Lean mass %^*^		52.5 (6.2)

Fasting glucose^∧^		4.8 (0.8)

Fasting insulin^∧&^		8.7 (6.0)

2 hr glucose^∧^		5.5 (2.8)

^*^Mean (standard deviation), independent samples *t*-test.

^∧^Median (interquartile range), Mann-Whitney *U* test.

^&^4 cases missing (1 intervention; 3 control).

**Table 2:** Change between 3-month and baseline measurements.

Characteristic	Intervention *N* = 11	Control *N* = 12	*P*-value
Weight^∧^ (kg)	−2.5 (1.4)	0.0 (2.3)	0.002
BMI^∧^ (kg/m^2^)	−0.9 (0.7)	0.0 (0.8)	0.002
Waist^∧^ (cm)	−3.0 (4.0)	0.5 (4.8)	0.037
Hip^∧^ (cm)	−3.0 (5.0)	0.0 (4.8)	0.006
Body fat %^∧&^	−1.1 (4.5)	−0.3 (1.3)	0.393
Lean mass %^∧&^	0.9 (3.3)	0.0 (2.6)	0.436
Fasting glucose^∧&^	0.3 (0.5)	−0.1 (0.6)	0.052
Fasting insulin^∧#^	−0.5 (2.4)	0.173	0.830
K 10 total score^∧^ (measure of distress and anxiety over the previous month)	0.0 (4.0)	1.0 (4.0)	0.193
WEL total score (measure of attitudes, feelings, and efficacy related to food and eating behaviours)	27.8 (20.1)	13.9 (37.4)	0.290
Negative emotions	5.5 (2.9)	3.5 (8.5)	0.472
Availability	7.1 (5.5)	1.0 (7.0)	0.036
Social pressure	6.1 (5.4)	4.1 (9.4)	0.545
Physical discomfort	4.5 (6.7)	4.5 (8.4)	0.978
Positive activities	4.6 (4.9)	0.9 (7.6)	0.188
HEPA	135 (225)	0 (418)	0.190
Fat	0.2 (0.4)	0.2 (0.5)	0.824
Fibre	−0.04 (0.8)	0.1 (0.4)	0.576
Total	0.1 (0.5)	0.2 (0.4)	0.682

All results are mean (standard deviation) unless otherwise stated.

^∧^Median (interquartile range), Mann-Whitney *U* test.

^&^3 cases missing (1 intervention; 2 control).

^#^5 cases missing (1 intervention; 4 control).

**Table 3:** Pedometer and nutrition workshop data.

Characteristic	Mean (SD)	Range
Number of times pedometer data uploaded	90 (31)	39–145
Number of days where steps were recorded	71 (31)	30–109
Number of steps per day	4,687 (3,510)	0–16,645
Number of times website messages accessed	28 (26)	3–74
Number of nutrition workshops attended	3 (1)	0–4

SD: standard deviation.
